# Temperature-controlled ablation of the mitral isthmus line using the novel DiamondTemp ablation system

**DOI:** 10.3389/fcvm.2022.1046956

**Published:** 2022-11-24

**Authors:** Laura Rottner, Ilaria My, Ruben Schleberger, Fabian Moser, Julia Moser, Paulus Kirchhof, Feifan Ouyang, Andreas Rillig, Andreas Metzner, Bruno Reissmann

**Affiliations:** ^1^Department of Cardiology, University Heart and Vascular Center Hamburg, Hamburg, Germany; ^2^Institute of Cardiovascular Sciences, University of Birmingham, Birmingham, United Kingdom; ^3^Deutsches Zentrum für Herz-Kreislauf-Forschung (DZHK) e.V. (German Center for Cardiovascular Research), Hamburg, Germany

**Keywords:** temperature-controlled mitral isthmus ablation atrial arrhythmias, atrial fibrillation ablation, DiamondTemp catheter, highpower short-duration, mitral isthmus line atrial arrhythmias, temperature-controlled ablation, mitral isthmus line

## Abstract

**Background:**

The novel DiamondTemp™ (DT)-catheter (Medtronic^®^) was designed for high-power, short-duration ablation in a temperature-controlled mode.

**Aim:**

To evaluate the performance of the DT-catheter for ablation of the mitral isthmus line (MIL) using two different energy dosing strategies.

**Materials and methods:**

Twenty patients with recurrence of atrial fibrillation (AF) and/or atrial tachycardia (AT) following pulmonary vein (PV) isolation were included. All patients underwent reisolation of PVs in case of electrical reconnection and ablation of a MIL using the DT-catheter. Application durations of 10 (group A, *n* = 10) or 20 s (group B, *n* = 10) were applied. If bidirectional block was not reached with endocardial ablation, additional ablation from within the coronary sinus (CS) was conducted.

**Results:**

In 19/20 (95%) patients, DT ablation of the MIL resulted in bidirectional block. Mean procedure and fluoroscopy time, and dose area product did not differ significantly between the two groups. In group B, fewer radiofrequency applications were needed to achieve bidirectional block of the MIL when compared to group A (26 ± 12 vs. 42 ± 17, *p* = 0.04). Ablation from within the CS was performed in 8/10 patients (80%) of group A and in 5/10 (50%) patients of group B (*p* = 0.34). No major complication occurred.

**Conclusion:**

Mitral isthmus line ablation with use of the DT-catheter is highly effective and safe. Longer radiofrequency-applications appear to be favorable without compromising safety.

## Introduction

Ablation of the mitral isthmus line (MIL) is a recognized strategy to treat perimitral reentrant tachycardia. MIL ablation may be also performed for substrat modification in patients suffering from persistent atrial fibrillation (AF) (1). MIL ablation with the endpoint of bidirectional conduction block is known to be challenging using conventional ablation catheters, and often requires not only extensive endocardial ablation, but also epicardial ablation from within the coronary sinus (CS) (2).

Newly, the DiamondTemp™ (DT) ablation system (Medtronic^®^, Inc., Minneapolis, MN, USA) was designed for high-power, short-duration ablation in temperature-controlled mode. The tip of the DT ablation catheter features six thermocouples at its tip, imbedded in a network of industrial diamonds which offer high thermal diffusivity allowing for low irrigation flow rates and a direct temperature feedback (3).

The use of the DT ablation system for pulmonary vein isolation (PVI) was proven to be effective and safe (3); however, data on substrate modification, i.e., creation of linear lesions, are lacking.

The aim of the present study was to evaluate the performance of the DT-catheter for ablation of the MIL applying two different energy dosing strategies.

## Materials and methods

### Patient cohort

The study population includes 20 consecutive patients with recurrence of symptomatic persistent AF and/or atrial tachycardia (AT) after PVI in a previous ablation procedure.

The following key exclusion criteria were predefined: a left atrial (LA) enlargement (LA-diameter > 60 mm), a left ventricular ejection fraction <30%, end-stage heart failure, severe valve regurgitation, or stenosis and/or contraindications to post-interventional oral anticoagulation.

### Preprocedural management

Prior to the procedure, transesophageal echocardiography was performed to rule out intracardiac thrombi. In patients on vitamin K antagonists, ablation was performed under therapeutic INR values of 2–3. In patients treated with novel oral anticoagulants (NOACs), anticoagulation was stopped the day before the procedure.

### Three-dimensional electroanatomic mapping

A 7F steerable decapolar catheter (7F Parahis, Biosense Webster, Diamond Bar, CA, USA) was placed in the CS. After conduction of double transseptal puncture guided by fluoroscopy and using a modified Brockenbrough technique, two SL1 sheaths (St. Jude Medical, MN, USA) were advanced into the LA (4, 5). Heparin was used to achieve and maintain an activated clotting time of >300 s. Selective PV angiographies were performed in order to identify the individual PV anatomy and ostia. A three-dimensional (3D) electroanatomic map of the LA was created using a multipolar mapping catheter in conjunction with the Ensite cardiac mapping system (NavX, Abbott, Inc., Chicago, IL, USA). The ipsilateral PV ostia were tagged on the 3D LA map according to angiographic and electrophysiological criteria.

### Ablation strategy

In all patients, the novel DT ablation system was used for RF-based point-by-point ablation. Our ablation strategy for RF-based AF-ablation has previously been described in detail (2, 6). In brief, electrical re-isolation of PVs was performed, if electrical reconnection of previously isolated PVs was detected *via* a circular mapping catheter (Advisor™, Abbott). A catheter-tip temperature limit of 60°C, a temperature-controlled power of 50 W, and an application duration of 10 s was therefore determined (7).

**FIGURE 1:**
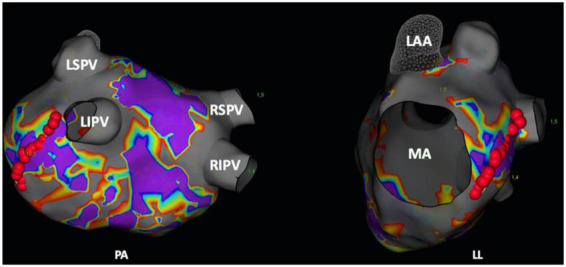
Postero-anterior (PA) and left-lateral (LL) view of a three-dimensional (3D) left atrial map (NavX, Abbott, Chicago, IL, USA). Red points delineate the ablation spots of the mitral isthmus line. LAA, left atrial appendage; LIPV, left inferior pulmonary vein; LSPV, left superior pulmonary vein; MA, mitral valve annulus; RIPV, right inferior pulmonary vein; RSPV, right superior pulmonary vein.

Subsequently, MIL ablation was performed either to treat perimitral atrial tachycardia or as substrate modification (Figure 1). According to our standard approach, a decapolar circular mapping catheter (Advisor™, Abbott) was placed inside the left atrial appendage (LAA), and a 7-F multipolar electrode catheter was positioned within the CS along the mitral annulus. Ablation of the MIL was performed by creating a line from the mitral annulus to the antero-inferior aspect of the wide circumferential lesion set encircling the left PVs. For MIL ablation a catheter-tip temperature limit of 60°C, a temperature-controlled power of 50 W with an application duration of either 10 s (group A, *n* = 10) or 20 s (group B, *n* = 10) was set. Patients were alternately assigned to one of the two treatment groups.

If conduction block of the MIL was not achieved with endocardial ablation only, epicardial ablation from within the CS was additionally performed. Therefore, a temperature limit of 60°C, a temperature-controlled power of 20 W and an application duration of 30 s was determined. Conduction properties across the mitral isthmus were continuously monitored during ablation. Bidirectional block of the MIL was defined as follows: (1) verification of widely spaced double potentials along the MIL during pacing from the LAA or from the distal CS; (2) a counter-clockwise activation along the mitral annulus (CS activation: proximal to distal) when pacing from the LAA; and (3) by use of differential pacing maneuvers (2).

If typical atrial flutter was additionally documented prior to or during the procedure, bidirectional block of the cavotricuspid isthmus was additionally performed. Durable PVI as well as conduction block of the MIL were ensured after a 30 min waiting period.

### Endpoints

The primary endpoint was bidirectional conduction block of the MIL as confirmed by mapping and pacing maneuvers.

Secondary endpoints included (1) the need for epicardial ablation from within the CS to achieve bidirectional block, (2) electrical reconnection across the mitral isthmus during repeat procedures, and (3) periprocedural complications.

### Postprocedural care

Our detailed postprocedural care strategy has been described previously (5, 7). After the procedure a transthoracic echocardiography was conducted to rule out pericardial effusion. Previously ineffective antiarrhythmic drugs (AAD) were prescribed for a further 3 months. All patients were treated with proton-pump inhibitors for 6 weeks after the procedure. In patients on vitamin K antagonists and an subtherapeutic INR < 2.0 low molecular-weight heparin was prescribed until a therapeutic INR of 2–3 was reached. Pre-existing medication with NOACs was re-initiated 6 h after the ablation procedure. Anticoagulation was continued for at least 3 months, and thereafter based on the individual CHA_2_DS_2_-VASc score.

According to our institutional standard a 12-lead ECG and Holter recordings were performed before discharge to confirm stable sinus rhythm. In patients with symptomatic arrhythmia recurrence, who were scheduled for a repeat ablation, conduction block across the previously deployed MIL was evaluated during the repeat ablation procedure (2).

### Statistical analysis

Continuous data are given as means and standard deviations, if the variables were normally distributed, or as medians, 25th and 75th percentiles otherwise. Categorical data are described with absolute and relative frequencies.

Continuous variables were compared using Student’s *t*-test or the Mann–Whitney U test. A Fisher exact test was performed to compare categorical variables. All *p*-values were two-sided. *P*-values < 0.05 were considered significant.

All calculations were conducted using R version 3.6.0 (2019).

## Results

### Patient characteristics

A total of 20 consecutive patients with symptomatic recurrence of persistent AF (19/20 [95%] patients) and/or perimitral atrial tachycardia (2/20 [10%] patients), who underwent repeat ablation procedure, were included. Repeat ablation included PV re-isolation (in case of electrical PV-reconnection) and MIL ablation using the DT ablation system.

In 6/10 (60%) patients of group A and in 8/10 (80%) patients of group B electrical reconnection of previously isolated PVs was documented. In 3/6 (50%) patients of group A with reconnection of at least one PV and in 3/8 (38%) patients of group B index-PVI was performed using the cryoballoon, whereas the remaining patients were treated by use of RF.

Detailed patient characteristics are given in Table 1.

**TABLE 1:** Baseline characteristics.

Parameter	Group A (10 s)	Group B (20 s)
Age, year	66 ± 8	73 ± 6
Sex (male), *n* (%)	8/10 (80)	6/10 (60)
Hypertension, *n* (%)	8/10 (80)	7/10 (70)
Diabetes mellitus, *n* (%)	0/10 (0)	0/10 (0)
CAD, *n* (%)	2/10 (20)	1/10 (10)
SHD, *n* (%)	5/10 (50)	3/10 (30)
LVEF, %	55 ± 5	56 ± 6
LA-diameter, mm	45 ± 3	46 ± 3
CHA_2_DS_2_-VASc score	2 [1; 3]	2 [1; 3]
**AAD at baseline**		
Beta blockers, *n* (%)	10/10 (100)	10/10 (100)
Class Ic AAD, *n* (%)	2/10 (20)	1/10 (10)
Class III AAD, *n* (%)	3/10 (30)	5/10 (50)
**Anticoagulation**		
Vitamin-K antagonist, *n* (%)	1/10 (10)	2/10 (20)
Direct oral anticoagulant, *n* (%)	9/10 (90)	8/10 (80)

Values are means ± standard deviations and frequencies (percentages) or medians with 25th, 75th percentiles. AAD, antiarrhythmic drugs; CAD, coronary artery disease; SHD, structural heart disease; LA, left atrium; LVEF, left ventricular ejection fraction.

### Procedural parameters and acute efficacy of mitral isthmus line ablation

Mean total procedure time, defined as time from femoral access until removal of vascular sheath, was 109 ± 26 min for group A and 104 ± 35 min for group B (*p* = 0.70). Fluoroscopy time and dose area product for group A and group B were 12 ± 4 vs. 13 ± 5 min (*p* = 0.39) and 451 [348; 658] vs. 487 [344; 572] cGycm^2^ (*p* = 0.65), respectively.

**FIGURE 2:**
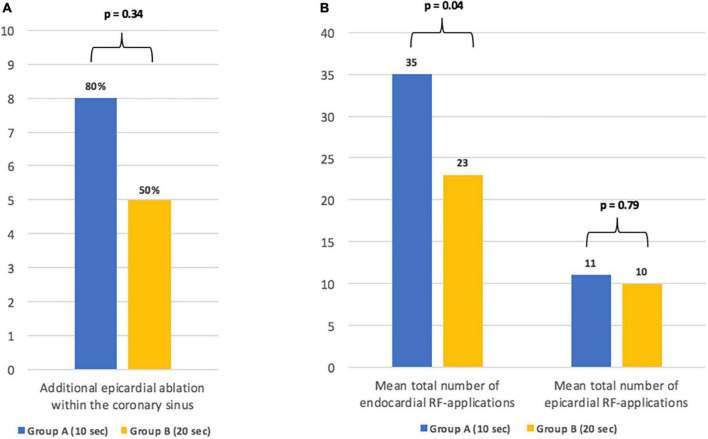
Details on the need for additional epicardial ablation **(A)** and the total number of endo- and epicardial radiofrequency-applications until bidirectional conduction block of the mitral isthmus line **(B)**.

In group B, bidirectional block of the MIL required fewer endocardial RF-applications when compared to group A (26 ± 12 vs. 42 ± 17, *p* = 0.04; Figure 2). Additional epicardial ablation was required in 8/10 patients (80%) of group A and in 5/10 (50%) patients of group B (*p* = 0.34; Figure 2).

Mean powers during endo- and epicardial ablation of the MIL were 49 ± 0.2 and 19 ± 1.4 W for group A, and 49 ± 0.4 and 19 ± 0.3 W for group B (*p* = 0.55 and *p* = 0.40). The corresponding mean temperatures were 47 ± 3 and 49 ± 6°C for group A, and 49 ± 3 and 50 ± 3°C for group B (*p* = 21 and *p* = 0.75).

There was no significant difference regarding the length of the MIL between the two groups (37 ± 7 mm for group A and 34 ± 9, *p* = 0.77).

Detailed procedural parameters are given in Table 2.

**TABLE 2:** Procedural data.

Parameter	Group A (10 s)	Group B (20 s)	*P*-value
Bidirectional block of MIL, *n* (%)	10/10 (100)	9/10 (90)	
Total procedure time, min	109 ± 26	104 ± 35	0.70
Total fluoroscopy time, min	12 ± 4	13 ± 5	0.39
Dose area product, cGycm	451 [348; 685]	487 [344; 572]	0.65
Total RF-applications until conduction block of the MIL	42 ± 17	28 ± 14	0.04
Epicardial ablation from within the coronary sinus, *n* (%)	8/10 (80)	5/10 (50)	0.34
Periprocedural complications, *n* (%)	0/10 (0)	1/10 (10)	

Values are means ± standard deviations and frequencies (percentages) or medians with 25th, 75th percentiles. A *p*-value < 0.05 is considered significant. CS, coronary sinus; MIL, mitral isthmus line; RF, radiofrequency.

### Safety

A pericardial effusion (<1 cm) without hemodynamic compromise, and with no need for pericardiocentesis, occurred in one patient of group B. In this patient MIL could not be blocked despite of extensive endocardial and epicardial ablation.

No other periprocedural adverse events occurred.

### Persistence of mitral isthmus line block in patients undergoing repeat procedures

Two out of twenty (20%) patients of group A had undergone repeat ablation due to symptomatic atrial arrhythmia. One patient presented with recurrence of AF and was planned to undergo catheter re-ablation after failure of AAD-therapy. During the procedure, durable bidirectional block of MIL could be documented. The second patient suffered from AT, which first occurred 4 months after MIL ablation. During the repeat procedure perimitral reentrant tachycardia was confirmed *via* entrainment and successfully treated with re-block of the MIL from epicardial.

## Discussion

The underlying study reports on acute efficacy and periprocedural safety of two different energy dosing strategies using the novel DT catheter for bidirectional conduction block of the MIL.

Main findings are:

(1) Mitral isthmus line ablation with use of the DT catheter is highly effective and safe;
(2) and longer RF-applications, as conducted in patients of group B, were associated with less endocardial ablation as well as less frequently additional epicardial ablation from within the CS without compromising safety.

### Technical aspects and current data on atrial fibrillation ablation using the DiamondTemp ablation system

Thermal feedback during energy delivery is highly desirable, because on the one hand irrevocable tissue injury requires a tissue temperature above 50°C (8), and on the other hand overheating with temperatures above 80°C results in char and thrombus formation, and is associated with an increased risk of steam pops (9, 10). Therefore, precise temperature detection during ablation is important to ensure creation of transmural lesions, while preventing periprocedural complications. However, catheter-tip temperature does not reflect the tissue temperature when using standard irrigated ablation catheters. To overcome these limitations, the novel DT ablation system was designed to re-establish the advantages of temperature-controlled RF ablation. The tip of the DT ablation catheter incorporates six externally located thermocouples and a network of industrial diamonds to rapidly shunt heat from the catheter tip allowing for precise temperature monitoring with low irrigation flow rates. During energy applications temperature is measured at these six thermocouples every 20 ms. Power is adjusted in real-time to maintain optimal tip-tissue temperature for durable lesion creation. Furthermore, the split-tip electrode provides real-time high-resolution electrograms and impedance recordings (3).

The use of the DT ablation system for PVI with determined ablation settings was proven to be effective and safe (7, 11, 12). Kautzner et al. (3) recently demonstrated that safety and efficacy of the DT ablation system was non-inferior to force-sensing RF ablation in a paroxysmal AF cohort. Moreover, in this study, shorter RF and LA dwell times, as well as less saline infusion was documented for the DT ablation group (3). These findings were confirmed by our study group using the DT ablation system for PVI (7).

### Ablation of the mitral isthmus line

Interventional therapy of AF with electric isolation of the PVs is recommended in symptomatic patients, and is associated with encouraging outcomes, especially in patients with paroxysmal AF (4, 13). However, long-term maintenance of sinus rhythm by catheter ablation is still challenging. Different ablation strategies have been applied to improve arrhythmia-free survival by expanding catheter ablation beyond PVI, particularly in patients with persistent AF (14). One option is ablation of the MIL, which may eliminate pro-arrhythmic triggers as well as substrate of AF, and prevent macro-reentrant LA tachycardia that occurs frequently in patients with repeat ablation of AF (15, 16).

The achievement of an acute bidirectional conduction block remains a challenge. A benefit of this additional substrate modification largely depends on completeness of deployed lines as incomplete lines may be pro-arrhythmic (17). Importantly, the STAR-AF II trial (head-to-head comparison of PVI alone vs. PVI plus ablation of complex fractionated electrograms vs. plus ablation of linear lines) found no difference regarding the arrhythmia-free survival for a PVI-alone ablation strategy vs. PVI plus additional substrate modification. However, in this study bidirectional block of the deployed linear lesions could only be achieved in 74% (18), which might be one explanation for the high rate of arrhythmia recurrences in this group.

Ablation of the MIL requires careful consideration of individual anatomical aspects, i.e., thickness of the myocardial tissue, distance between the mitral annulus and the lateral PVs, as well as location of epicardial vessels such as the left circumflex artery (19, 20). Moreover, the myocardial tissue is thickest at the inferior aspect of the mitral isthmus, while it significantly decreases toward the superior aspect along the posterior base of the LA appendage (21). As a consequence, superolateral ablation of the MIL is associated with higher acute success rates when compared to conventional ablation at the inferior aspect of the MIL, but it may increase procedural complication (2).

The rate of acute conduction block of the MIL ranges between 64 and 74% (18, 20). In our study, bidirectional block of the mitral isthmus line was achieved in 19/20 (95%) patients by exclusive ablation of a conventional inferolateral MIL. Importantly, the use of the DT catheter for MIL ablation resulted in a relevant reduction of the procedure time when compared to previously published data of our study group with conventional non-contact force-sensing catheters (2). In addition, the total number of RF applications, and the need for additional epicardial ablation was significantly reduced when applying longer RF-applications of 20 s. For PVI the DT catheter demonstrated high efficacy by use of 10 s per RF-application (7), but longer energy application appears to be favorable when targeting the MIL. However, the risk for steam pops might be underestimated by the limited number of patients of the present study. It is all the more important to be cautious for contact and impedance drop when applying longer RF-applications.

### Strength and limitations

Although the current study comprised only a small patient cohort, this is the first study to report on the performance of the DT catheter during ablation of the MIL comparing two different energy dosing protocols. The design of the underlying study does not allow for conclusions on the impact of bidirectional conduction block of the MIL on the arrhythmia-free survival. More data are needed to confirm our findings.

## Conclusion

Mitral isthmus line ablation with use of the novel DT ablation system is feasible, highly effective, and safe. Longer RF-applications resulted in a reduced number of total RF applications, and a less frequent need for additional epicardial ablation from within the CS.

## Data availability statement

The original contributions presented in this study are included in the article/supplementary material, further inquiries can be directed to the corresponding author.

## Ethics statement

The study involving human participants was reviewed and approved by the Ethikkommission Hamburg (2022-300214_WF) and was conducted in accordance with the Declaration of Helsinki of 2013. The patients/participants provided their written informed consent to participate in this study.

## Author contributions

LR and BR conceived of the presented idea, verified the analytical methods, and performed statistical analyses. LR was mainly responsible for the data collection. FO, AR, and AM supervised the findings of this work. All authors discussed the results and contributed to the final manuscript.
